# Effects of Ascorbic Acid on Serum Level of Unconjugated Estriol and Its Relationship with Preterm Premature Rupture of Membrane: A Double-Blind Randomized Controlled Clinical Trial

**Published:** 2013-09

**Authors:** Mehrangiz Zamani, Mohammad Taghi Goodarzi, Navaz Sadat Lavasani, Abas Khosravi

**Affiliations:** 1 Department of Obstetrics and Gynecology, Hamadan University of Medical Sciences, Hamadan, Iran;; 2 Research Center for Molecular Medicine, Hamadan University of Medical Sciences, Hamadan, Iran;; 3 Production Manager, Modava Pharmaceutical Co., Tehran, Iran

**Keywords:** Unconjugated, Estrio, Vitamin C

## Abstract

**Background: **Vitamin C is a water-soluble antioxidant that not only stimulates and protects collagen synthesis but also plays an important role in maintaining cellular integrity in a normal pregnancy. This study surveyed the effects of ascorbic acid on the serum level of unconjugated estriol and the relationship between unconjugated estriol and preterm premature rupture of membrane (PPROM).

**Methods: **This double-blind, randomized clinical trial recruited 60 patients with predisposing factors to PPROM. The women were randomly divided into two groups of intervention and control and received vitamin C and placebo, respectively. The intervention group received 250 mg vitamin C twice a day and the controls received the placebo only. Unconjugated estriol was measured using the ELISA. All data were extracted and recorded in a checklist and compared using descriptive statistics as well as the *x*^2^, Fisher exact, and *t* tests.

**Results:** The demographic data showed no difference between the two groups. The mean level of serum unconjugated estriol was significantly lower in the intervention group than in the control group (P=0.044). Also, the frequency of PPROM was lower in the intervention group, but the difference was not significant (P>0.05). Unconjugated estriol levels were not significantly different between the healthy women and the PPROM patients.

**Conclusion:** This study demonstrated that vitamin C administration decreased unconjugated estriol levels in the patients with PPROM. The findings of this study also indicated that administration of ascorbic acid was a safe and effective method to reduce the incidence of PPROM. Alteration in unconjugated estriol is an active mediator for this effect.

## Introduction

Premature rupture of membrane (PROM) is the rupture of the chorioamniotic membrane and leakage of the amniotic fluid before delivery contractions.^1^ PROM is the commonest cause of premature delivery. Recent studies have reported that with occurrence rates of 6 to 19%, PROM is the leading cause of mortality in the prenatal period.^2^ Preterm PROM (PPROM), which leads to PROM before the 37^th^ week of pregnancy, is responsible for 40 to 50% preterm deliveries and necessitates hospitalization in the neonatal intensive care unit (NICU).^3^

Various causes have so far been propounded for PPROM – with a sizable bulk of evidence relating it to biochemical processes such as disorders of collagen synthesis in the extra-cellular matrix of amnion and chorion and planned death of cells in fetal tissues. It is suspected that mediators released from stretching membrane or infection and activation of destructive enzymes in the matrix lead to the rupture of the uterus or amniotic membranes.^4^

One of the factors involved in the activation of membrane destruction is the activity of reactive oxygen species (ROS). Because antioxidants suppress ROS by their chemical characteristic, consumption of materials like ascorbic acid or vitamin C is effective in the stability of the membrane and prevention of PROM and PPROM.^5^ Epidemiological studies, linking clinical conditions known to produce ROS or reduce antioxidant protection to PPROM, support this hypothesis.^6^ Further evidence in this field comes courtesy of in vitro studies in which membrane segments exposed to ROS exhibited tissue changes consistent with PPROM.^6^ Also, excessive collagen degradation in chorioamnion and amniotic samples from PPROM patients has been previously demonstrated.^6^

Vitamin C, in addition to its antioxidant role, not only is an important factor in the synthesis of collagen but also controls the expression of type IV collagen gene.^7^^,^^8^ This assumption is in agreement with findings like the increased likelihood of PPROM as a consequence of smoking, which is a source of ROS.^9^

Maintaining cellular integrity in a normal pregnancy needs the inhibition of peroxidation reactions, which is important to protect proteins, enzymes, and cells from destruction by peroxides.^10^ Antioxidant defense mechanisms contain both enzymes such as superoxide dismutase and free radical scavengers such as vitamin C. 

Because vitamin C is not synthesized in humans, its consumption is necessary for the prevention of scurvy, which accompanies weakness of the collagen system. Vitamin C is the cofactor for enzymes like lysyl hydroxylase and prolyl hydroxylase, enzymes that are very important for making hydroxylysine and hydroxyproline, which play a crucial role in the stability of the structure of collagen triple helix.^11^

Predicting the probability of PROM and PPROM is of vital importance. Therefore, researchers have devised and assessed a vast array of clinical and paraclinical methods in search of an optimal modality. One of these methods is measuring the estriol level in serum or saliva. This assumption is based on the increase in the mother’s estriol.^12^ Estriol appears in the 9^th^ week of pregnancy and rises gradually with the growth of the fetus. This increase is accompanied by a rise in steron and estradiol levels; however, estriol continues to increase until delivery – while steron and estradiol exhibit no clear changes after the 34^th^ week of pregnancy.^13^

Oxidative stress is known as a key feature in PROM.^12^ One study reported that antioxidant therapy conferred protection against hypochlorous acid-induced damage and concluded that PROM was, in part, due to ROS and antioxidant deficit, which resulted in membrane damage.^14^ Vitamin supplementation, including vitamin C, can prevent oxidative stress and consequently lower the risk of PROM.^14^

Estriol and afertrin are produced late in pregnancy by fetus germination. Estriol enters from the fetus membrane into the mother’s circulation and immediately transforms to sulfate and glucuronide, which can be removed easily. Unconjugated estriol (UEs) transforms in the mother’s liver to sulfate and glucuronide and is repelled by urine with a half life of 20 to 30 minutes. In the mother’s circulation, UEs accounts for up to 10% of total estriol. 

Because UEs is not affected by liver and kidney diseases as well as antibiotics and because conjugated estriol has a short half life, only UEs was selected to be measured in this study. The aim of this study was firstly to examine the effect of vitamin C supplementation on the serum level of UEs and secondly to determine any possible correlation between vitamin C administration and the presence of PPROM. 

## Patients and Methods

**Figure 1:**
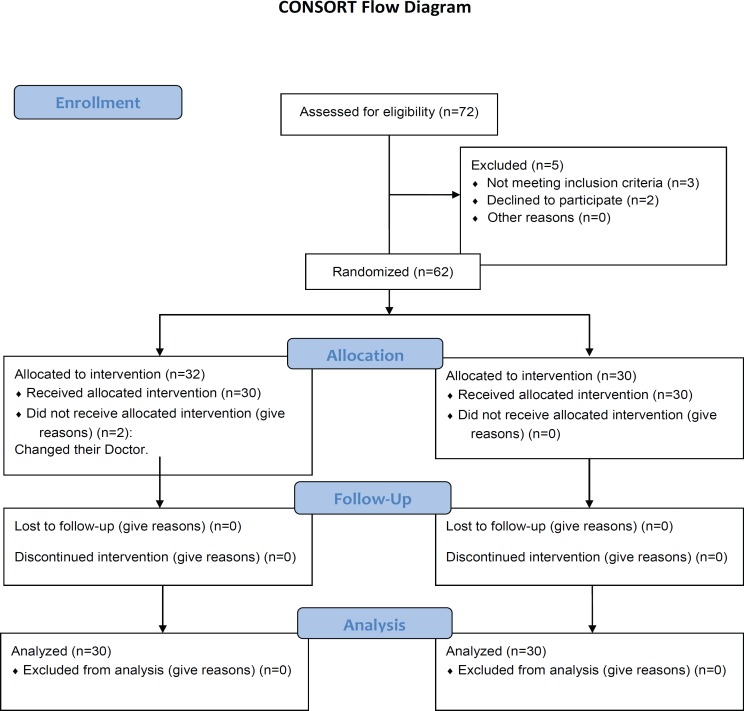
Study flow diagram of pregnant women recieving vitamin C or placebo to assess preterm premature rupture of membrane

The present study was approved by the Ethics Committee of Hamadan University of Medical Sciences, and was registered with the Government Database for Clinical Trials (reference no: IRCT201012083580N3). This double-blind, randomized, controlled clinical trial recruited 60 pregnant women with the age range of 20-40 years, referring to Fatemieh Hospital and Shaykhoraies Clinic (Hamadan, Iran). Figure 1 summarizes the study flow diagram. 

All the studied subjects were selected from pregnant women at high risk of PPROM and in the 18^th^ week of pregnancy, as confirmed by sonography. The inclusion criteria included history of previous PROM and PPROM, and the exclusion criteria encompassed consumption of medication in defined intervals, history of uterus surgery, history of Cesarean section, short cervix, smoking, and pregnancy with artificial vaccination. PPROM was diagnosed via the Fern test and sonography. Cases diagnosed with specific disorders such as infection and delivery contractions during the survey were excluded from the study. To rule out infectious cases, vaginal examination was carried out and also the sign and symptoms of chorioamnionitis were evaluated. 

Written consent was obtained from all the patients. The study population was randomly divided into two equally-sized intervention and control groups-based on the table of random numbers via a blind method. 

The medicine and placebo were prepared in the same bottles and blinded by code. The matrix compounds of the placebo and vitamin C tablets were identical and were made by the same company. None of the prescribing persons and patients was aware of the prescribed medicine. In the intervention group, 250 mg supplement of vitamin C (Modava Co., Iran) was prescribed twice a day (500 mg a day) and the control group received placebo with the same procedure. Length of the treatment period was until the 28^th^ week of pregnancy. The serum level of UEs was measured using the ELISA method (IBL, Germany), accompanied by blood sugar and hemoglobin assay. All the subjects were thereafter followed up to delivery and were evaluated for the incidence of PPROM. Finally, the results were analyzed using SPSS software (version 13.0) and the Student *t*-test, chi-square test, and Fisher exact test. A P value less than 0.05 was considered significant. 

## Results

The mean±SD of age in the intervention and control groups was 24.90±5.63 and 24.60±5.53 years, respectively. In the intervention group, 11 (36.6%) cases were nulliparous and 19 (63.6%) cases were multiparous, while in the control group, there were 8 (26.2%) nulliparous and 22 (73.4%) multiparous women. 

As is shown in table 1, there were no significant differences in terms of age and parity between the two studied groups. 

**Table 1:** Comparison of the demographic characteristics between the women receiving vitamin C or placebo

**P value**	**Parity**		**Mother`s Age (year)**	**Group**
	**Multi** **n (%)**	**Null** **n (%)**		
0.805	19 (63.4)	11 (36.6)	24.90±5.63	Vitamin C
0.297	22 (73.4)	8 (26.2)	24.60±5.53	Control

The serum UEs levels (mean±SD) were 16.81±9.98 and 22.21±11.03 ng/ml in the intervention and control subjects, respectively (table 2). A comparison of the serum UEs levels between the two studied groups showed a significantly lower level in the women who received vitamin C (P=0.044). The frequency of PPROM was lower in the group receiving vitamin C than in the control group, with the difference not constituting statistical significance (table 3). The UEs level in the PPROM-negative women (regardless of vitamin C consumption) was lower than that of the PPROM-positive subjects; nevertheless, the difference was not significant (19.02±11.01 and 22.98±8.60 ng/dl, respectively).

**Table 2:** Comparison of unconjugated estriol levels between the women receiving vitamin C and the control group in the 28th week of pregnancy

**P value**	**Unconjugated estriol ng/ml**	**Group**
0.044	22.21±11.03	Control
	16.81±9.98	Vitamin C

**Table 3:** Comparison of the frequency of PPROM between the group receiving vitamin C and the control group

**P value**	**PPPOM Frequency**		**Group**
	**Negative** **n (%)**	**Positive ** **n (%)**	
0.213	28 (93.3)	2 (6.7)	Vitamin C
	25 (83.3)	5 (16.7)	Control

## Discussion

The main cause of PROM with different pathologies is disorder in collagen metabolism. Reduction in the collagen content of embryo membranes decreases their stability and causes rupture, which has several side effects for both mother and embryo. 

Consumption of vitamin C during pregnancy can prevent PROM because it can regulate the metabolism of membranes’ collagen and augment their resistance. Vitamin C can also prevent an early increase in estriol during pregnancy. In this study, estriol levels were significantly lower in the patients receiving vitamin C than in the placebo group (P=0.044). The relative frequency of PPROM was also higher in the control group than in the intervention group; however, the difference was not statistically significant. A recent study demonstrated that plasma vitamin C was lower in women with PPROM; it concluded that a low plasma vitamin C concentration might be an associated risk factor for PPROM.^15^ Elsewhere, it was proposed that generation of ROS could be a potentially reversible pathophysiological pathway that might lead to PPROM.^9^ Accordingly, consumption of antioxidants such as vitamin C may lessen the risk of PPROM.

Our literature review of relevant studies did not yield any investigation measuring the relationship between serum estriol levels in PPROM and vitamin C consumption. Be that as it may, some studies have considered these factors separately. In 2003, Tejero et al.^16^ measured the concentration of vitamin C in leukocytes and found lower levels in the PROM patients than in the control group. Also, Plessinger et al.^14^ argued that foods were not substantial enough resources to provide the appropriate level of vitamins C and E, required for the prevention of PPROM, and suggested food supplements to compensate for such insufficiencies. Borna et al.^17^ studied 60 patients and observed that vitamin C, accompanied by vitamin E, increased the latency period. In 2003, Siega-Riz et al.^18^ suggested that vitamin C be incorporated in the protocol for pregnant women. In contrast to these findings and what is expected theoretically, Spinnato JR et al.^19^ reported that supplementation of vitamins C and E in a combination dose might be associated with a higher risk of PPROM and PROM. 

As regards measuring estriol, Heine et al.^20^ in a three-way blind study in 8 medical centers in the US measured oral estriol in 601 patients and claimed that it was a thorough method for predicting PROM.^20^ Goodwin^21^ in a review study concluded that a high estriol level was a risk factor for PROM and PPROM. 

In the present study, the maximum dose of vitamin C in the intervention group was 500 mg daily, which is considerably different from the amount determined by the US Health Organization (2000 mg). As a result, apropos of the side effects of the medicine, there was no risk to our study population. 

Two significant limitations of the present study are its use of a single dose of vitamin C and its relatively small sample size. Further studies are required to evaluate the effect of the different doses of vitamin C. It is also worthy of note that since concentrations of estrogen, estradiol, and estriol in the mother’s saliva are a reflection of unconjugated serum levels and free concentrations of these compounds in pregnancy,^22^ it is possible to use saliva for the assessment of these hormones. 

## Conclusion

Based on the results of the present study, it can be concluded that consumption of vitamin C may decrease the serum level of UEs in PPROM patients, which can be considered as an index in reducing the probability of PROM or PPROM. 

The findings of this study also indicated that administration of ascorbic acid was a safe and effective method to reduce the incidence of PPROM. Alteration in UEs is an active mediator for this effect.
